# Performance of Large Language Models in the Japanese Public Health Nurse National Examination: Comparative Cross-Sectional Study

**DOI:** 10.2196/82842

**Published:** 2026-02-20

**Authors:** Yutaro Takahashi, Ryota Kumakura, Rie Okamoto, Shizuko Omote

**Affiliations:** 1 Faculty of Health Sciences, Institute of Medical, Pharmaceutical and Health Sciences, Kanazawa University, Kodatsuno 5-11-80, Kanazawa, Ishikawa, 920-0942, Japan, 81 76-265-2559

**Keywords:** large language models, public health nursing, licensure, nursing, artificial intelligence, AI, education

## Abstract

**Background:**

Large language models (LLMs) have shown promising results on Japanese national medical and nursing examinations. However, no study has evaluated LLM performance on the Japanese Public Health Nurse National Examination, which requires specialized knowledge in community health and public health nursing practice.

**Objective:**

This study aimed to compare the performance of multiple LLMs on the Japanese Public Health Nurse National Examination and evaluate their potential utility in public health nursing education.

**Methods:**

Three LLMs were evaluated: GPT-4o, Claude Opus 4, and Gemini 2.5 Pro. All 110 questions from the 111th Public Health Nurse National Examination were administered using standardized prompts. Questions were classified by format (text vs figure or calculation), content (general vs situational), and selection type (single vs multiple choice). Accuracy rates and 95% CIs were calculated, with statistical comparisons performed using chi-square tests.

**Results:**

All LLMs exceeded the passing criterion (60%). The accuracy rates were as follows: 85.5% (94/110) for GPT-4o (95% CI 77.5%‐91.5%), 91.8% (101/110) for Claude Opus 4 (95% CI 85.0%‐96.2%), and 92.7% (102/110) for Gemini 2.5 Pro (95% CI 86.2%‐96.8%). No significant differences were found among the LLMs (*P*>.99). However, all models showed lower accuracy on multiple-choice questions than on single-choice questions, with significant intramodel differences observed for GPT-4o (10/16, 62.5% vs 82/92, 89.1%; *P*=.01) and Claude Opus 4 (12/16, 75% vs 87/92, 94.6%; *P*=.03).

**Conclusions:**

LLMs demonstrated high performance on a public health nursing examination but showed limitations in complex reasoning requiring multiple-choice selection. These findings suggest the potential for LLM use as educational support tools while highlighting the need for cautious implementation in specialized nursing education.

## Introduction

With advances in artificial intelligence (AI), large language models (LLMs) have gained attention in various fields. High-performance LLMs have been developed, including GPT-3.5, GPT-4, Anthropic’s Claude, and Google’s Gemini [1-3], which have demonstrated their ability to generate contextually appropriate responses to complex questions. In Japan, the capabilities of these models have been evaluated using questions from the national medical and nursing examinations. Liu et al [4] compared GPT-4o, GPT-4, Claude Opus 3, and Gemini 1.5 Pro using Japan’s national medical examinations and found that GPT-4o achieved the highest accuracy rate (89.2%) and intermodel performance depending on the subject area and question format. Takagi et al [5] also found that GPT-4 significantly outperformed GPT-3.5 in national medical examinations (79.9% vs 50.8%), revealing significant performance gaps between model generations. Taira et al [6] reported that LLM accuracy declined for questions about pharmacology, social welfare, and related legal regulations on national nursing examinations, suggesting limitations in responding to questions requiring specialized knowledge and institutional understanding. These findings demonstrate LLM utility and associated challenges in Japanese language processing and professional examinations.

However, no study has compared the performance of multiple LLMs on the Japanese Public Health Nurse National Examination, which assesses the knowledge and skills necessary for public health nursing practice. The Public Health Nurse National Examination comprises content addressing community-based health issues, multidisciplinary collaboration, and public health nursing activities, requiring not only medical knowledge but also an understanding of Japan’s community health systems [7]. Compared to clinical medicine, public health nursing presents unique challenges for LLMs. While medical examinations primarily assess biomedical knowledge and clinical reasoning, public health nursing requires the integration of social determinants of health, public health policy knowledge, community-level interventions, and understanding of local health systems. These multifaceted aspects demand complex reasoning that simultaneously considers multiple factors, which may pose greater challenges for current LLMs. Therefore, this study aimed to compare and evaluate the performance of GPT-4o (Open AI), Claude Opus 4 (Anthropic), and Gemini 2.5 Pro (Google AI) on questions from the Japanese Public Health Nurse National Examination to clarify the extent to which AI can respond to the specialized knowledge and skills necessary for public health nursing. We believe our work has significance for examining the potential for AI use in future public health nursing education.

## Methods

### Study Design

This comparative cross-sectional study evaluated the performance of 3 LLMs on the Japanese Public Health Nurse National Examination. We used a census sampling approach, analyzing all questions from the examination to compare accuracy rates across different LLMs and question types.

### Tested LLMs

Three representative LLMs were selected: OpenAI’s GPT-4o [1], Anthropic’s Claude Opus 4 [2], and Google’s Gemini 2.5 Pro [3].

### Japanese Public Health Nurse National Examination

The Japanese Public Health Nurse National Examination is conducted based on the Act on Public Health Nurses, Midwives, and Nurses to assess the knowledge and skills necessary for public health nurses [8]. It includes 110 questions. The questions are classified as general and situational. General questions are worth 1 point each, whereas situational questions are worth 2 points each. The passing criterion is 60% correct answers for general and situational questions combined, although the percentage may be adjusted when inappropriate questions are excluded. The questions are in multiple-choice format, and the participants must select one or more correct answers from 4 to 5 options. The pass rate is 90% to 95%.

### Study Population and Question Selection

This study used a census sampling approach, including all 110 questions from the 111th Public Health Nurse National Examination administered in February 2025 [9]. No sampling was conducted as the entire population of examination questions was analyzed. For questions containing figures and tables, figures were processed as image data, whereas the question text was handled as text. No questions were designated as inappropriate by the Ministry of Health, Labour and Welfare; the passing criterion was 60% [9]. The examination questions were classified according to format (text questions and figure or calculation questions), content (general and situational questions), and selection type (single- and multiple-choice questions).

### Prompt Engineering

Because prompt engineering significantly affects the generated output, the question input formats were standardized. On the basis of previous research [4-6], 6 prompts corresponding to different types of questions were created (Table 1).

**Table 1.** Standardized prompts for different question types in the Japanese Public Health Nurse National Examination. Template placeholders (“<” and “>”) indicate where specific content was inserted for each question type.

Prompt type	Prompt template
Prompt 1: general questions	“Japanese Public Health Nurse National Examination questions are presented. Please answer the following question in brief by selecting an option. Select one option unless otherwise specified. Question: <Question content> 1. <Option 1> 2. <Option 2> 3. <Option 3> 4. <Option 4> 5. <Option 5> (if applicable)”
Prompt 2: situational questions	“Japanese Public Health Nurse National Examination questions are presented. Based on the following situational setting, please answer the question in brief by selecting an option. Select one option unless otherwise specified. Situation: <Situational content> Question: <Question content> 1. <Option 1> 2. <Option 2> 3. <Option 3> 4. <Option 4> 5. <Option 5> (if applicable)”
Prompt 3: image questions	“Japanese Public Health Nurse National Examination questions are presented. Please review the following image and answer the question in brief by selecting an option. Select one option unless otherwise specified. Question: <Question content> 1. <Option 1> 2. <Option 2> 3. <Option 3> 4. <Option 4> 5. <Option 5> (if applicable)”
Prompt 4: situational questions with images	“Japanese Public Health Nurse National Examination questions are presented. Based on the following situational setting, please review the image and answer the question in brief by selecting an option. Select one option unless otherwise specified. Situation: <Situational content> Question: <Question content> 1. <Option 1> 2. <Option 2> 3. <Option 3> 4. <Option 4> 5. <Option 5> (if applicable)”
Prompt 5: calculation questions	“Japanese Public Health Nurse National Examination questions are presented. Please read the following question, review the mark sheet format in the image, and answer the question in brief by selecting an option. Provide your answer as a numerical value in brief. Question: <Question content>”
Prompt 6: situational calculation questions	“Japanese Public Health Nurse National Examination questions are presented. Please read the following situational setting and question, review the mark sheet format in the image, and answer the calculation question. Provide your answer as a numerical value in brief. Situation: <Situational content> Question: <Question content>”

### Data Collection Procedures

Questions were input from June 25 to 26, 2025. The question text and images were directly inserted into each LLM’s chat window. Each question was input in a new independent chat window to avoid potential influence from previous responses. All questions were administered in Japanese using standardized prompts.

The definition of “correct” answers was based on the official answers published by the Ministry of Health, Labour, and Welfare [9]. Only answers that clearly matched the official correct answers and followed the instructions provided in the question text were considered “correct.” Ambiguous answers, evident mistakes, unclear responses, and responses with an excessive number of answer choices were considered incorrect. All responses from the LLMs were independently reviewed and scored by 2 authors (YT and RK), with any discrepancies resolved through discussion.

### Data Analysis

For each LLM, the number of correct answers, accuracy rates, 95% CIs, total scores, and score rates were calculated. Accuracy rates were compared across question format (text vs figure or calculation), question content (general vs situational), and selection type (single vs multiple choice) both between LLMs (inter-LLM comparison) and within each LLM (intra-LLM comparison). Numerical input calculation questions were excluded from the selection type analysis.

Statistical comparisons were performed using chi-square tests for expected cell frequencies of ≥5 and the Fisher exact test when expected cell frequencies were <5. For multiple pairwise inter-LLM comparisons, the Bonferroni correction was applied to control for type I error. Statistical significance was set at *P*≤.05 (2 tailed). All statistical analyses were conducted using Stata (version 18.0; StataCorp LLC).

### Ethical Considerations

Ethics approval was not required because only data from a published database were analyzed.

## Results

Detailed results are presented in Table 2. The accuracy rates for each LLM for all 110 questions were as follows: 85.5% (n=94) of the questions for GPT-4o (95% CI 77.5%‐91.5%), 91.8% (n=101) of the questions for Claude Opus 4 (95% CI 85.0%‐96.2%), and 92.7% (n=102) of the questions for Gemini 2.5 Pro (95% CI 86.2%‐96.8%). The corresponding scores were 86.2% (125/145), 91.7% (133/145), and 93.1% (135/145), respectively, all of which exceeded the passing criterion (60%). In terms of question characteristics, the accuracy rates for general questions (n=75) were as follows: 84% (n=63) for GPT-4o, 92% (n=69) for Claude Opus 4, and 92% (n=69) for Gemini 2.5 Pro. The corresponding rates for situational questions (n=35) were 88.6% (n=31), 91.4% (n=32), and 94.3% (n=33), respectively.

**Table 2.** Performance of large language models (LLMs) on the 111th Public Health Nurse National Examination.

Category	GPT-4o		Claude Opus 4		Gemini 2.5 Pro		LLM comparison *P* value		
	Correct answers, n (%)	*P* value	Correct answers, n (%)	*P* value	Correct answers, n (%)	*P* value	GPT-4o vs Claude Opus 4	GPT-4o vs Gemini	Claude Opus 4 vs Gemini
Overall accuracy rate (n=110)	94 (85.5)	—^a^	101 (91.8)	—	102 (92.7)	—	>.99	>.99	>.99
Overall score (n=145)	125 (86.2)	—	133 (91.7)	—	135 (93.1)	—	>.99	>.99	>.99
By question content		.53		>.99		>.99			
General questions (n=75)	63 (84.0)		69 (92.0)		69 (92.0)		>.99	>.99	>.99
Situational questions (n=35)	31 (88.6)		32 (91.4)		33 (94.3)		>.99	>.99	>.99
By question format		.69		>.99		.60			
Text questions (n=98)	84 (85.7)		90 (91.8)		90 (91.8)		>.99	>.99	>.99
Figure or calculation questions (n=12)	10 (83.3)		11 (91.7)		12 (100.0)		>.99	>.99	>.99
By selection type		.04		.03		.09			
Single-choice questions (n=92)	82 (89.1)		87 (94.6)		87 (94.6)		>.99	>.99	>.99
Multiple-choice questions (n=16)	10 (62.5)		12 (75.0)		13 (81.3)		>.99	>.99	>.99

a Not applicable.

In terms of question format, the accuracy rates for the text questions (n=98) were as follows: 85.7% (n=84) for GPT-4o, 91.8% (n=90) for Claude Opus 4, and 91.8% (n=90) for Gemini 2.5 Pro. The corresponding rates for figure or calculation questions (n=12) were 83.3% (n=10), 91.7% (n=11), and 100% (n=12), respectively. The accuracy rates for single-choice questions (n=92) were as follows: 89.1% (n=82) for GPT-4o, 94.6% (n=87) for Claude Opus 4, and 94.6% (n=87) for Gemini 2.5 Pro. However, for the multiple-choice questions (n=16), all LLMs showed decreased accuracy, and the corresponding rates were 62.5% (n=10), 75% (n=12), and 81.3% (n=13), respectively.

Statistical comparisons among the LLMs showed no significant differences. Intra-LLM comparisons revealed significant differences between single- and multiple-choice questions for GPT-4o (*P*=.01) and Claude Opus 4 (*P*=.03), with the accuracy rates for multiple-choice questions being significantly lower.

## Discussion

### Principal Findings

This study compared and evaluated the performances of multiple LLMs on the Japanese Public Health Nurse National Examination. All the LLMs significantly exceeded the passing criterion of 60%. These results indicate that LLMs have acquired considerable specialized knowledge, with the performance of GPT-4o (94/110, 85.5%) being comparable to that of LLMs in previous medical examinations [4] and superior to that of older-generation models [6].

While all LLMs showed high overall accuracy rates, a clear performance decline was observed for multiple-choice questions. This contrasts with the high accuracy rates for single-choice questions, with significant differences found for GPT-4o and Gemini 2.5 Pro. This phenomenon demonstrates the current limitations of LLMs in complex reasoning, which requires the simultaneous evaluation of multiple concepts. As public health nursing practice requires comprehensive judgment considering multiple factors such as regional characteristics, residents’ needs, social resources, and multidisciplinary collaboration, the performance decline on multiple-choice questions indicates that LLMs have limitations in the complex decision-making faced in actual public health nursing practice.

The accuracy rates of the LLMs evaluated in this study (94/110, 85.5% to 102/110, 92.7%) and their scores (125/145, 86.2% to 135/145, 93.1%) substantially exceeded the passing standard (60%). The pass rate for the 111th Public Health Nurse National Examination was 94% [9]; however, this represents the proportion of examinees who exceeded the passing standard, and the overall mean score for all examinees is not publicly available. Therefore, a direct comparison between the overall academic performance of examinees and LLM performance is challenging.

In the field of medical education, multiple studies comparing LLM and student performance have been reported. A study comparing final-year emergency medicine students with AI models [10] demonstrated that students achieved a 79.4% accuracy rate, outperforming ChatGPT (72.5%) and Gemini (54.4%). The superiority of students was particularly pronounced in image-based questions, highlighting current limitations in AI models’ visual information processing capabilities. Additionally, a study using 1070 medical imaging questions [11] found that GPT-4 correctly answered 67.8% of the questions it attempted, whereas the students’ passing mean was 63%. However, the student majority vote achieved a 94.5% accuracy rate, substantially surpassing the AI. This demonstrates that even when individual students’ abilities may be equal to or slightly inferior to those of AI, collective student judgment significantly exceeds AI performance.

In our study, while LLM performance on figure or calculation questions was high (10/12, 83.3% to 12/12, 100%), the small number of questions (n=12) necessitates larger-scale validation. More importantly, the learning processes of LLMs and humans are fundamentally different. LLMs learn patterns from large volumes of text data, whereas public health nurses acquire decision-making capabilities by integrating practical experience with theoretical knowledge. Furthermore, human public health nurses possess essential practical competencies that are not measurable through written examinations, including ethical judgment, empathy, and interpersonal communication skills. As these previous studies [10,11] demonstrate, while AI shows potential as a supplementary educational tool, it cannot replace human capabilities, particularly in areas requiring visual interpretation, clinical reasoning, and collective judgment. Therefore, LLMs should be appropriately positioned as educational and learning support tools rather than as replacements for human public health nurses.

The results of this study have important implications from the perspective of competency development in public health nursing education. The “Practical Competencies Required of Public Health Nurses and Achievement Goals and Levels at Graduation” document by the Japanese Ministry of Health, Labour and Welfare [12] classifies public health nurse competencies into five domains: (1) ability to clarify community health issues and develop plans; (2) ability to provide continuous support and collaborative organizational activities for individuals, families, groups, and organizations to enhance community health promotion capacity and evaluate these activities; (3) community health crisis management capacity; (4) ability to develop projects, policies, social resources, and systems to enhance community health levels; and (5) professional autonomy and continuous quality improvement capacity. LLMs may be particularly effective in providing learning support during information gathering and assessment stages within domain 1. This competency includes information collection for clarifying community health issues, community diagnosis, and prioritization of health issues, where LLMs are expected to play a supplementary role in confirming foundational knowledge and organizing information.

For domain 1, the achievement level at graduation is set at either level 1 (“able to implement independently with minimal guidance”) or level 2 (“able to implement under supervision [from supervising public health nurses or faculty]”) [12]. As revealed in this study, LLMs demonstrated a performance decline in multiple-choice questions and have limitations in complex judgment tasks. Therefore, when using LLMs as educational tools, it is crucial to cultivate students’ ability to critically evaluate LLM outputs and maintain practical judgment skills based on community characteristics to reach these achievement levels.

Several ethical considerations must be addressed when using LLMs in public health nursing education. The Japanese Ministry of Education, Culture, Sports, Science and Technology guidelines [13] indicate that directly using generative AI outputs does not deepen students’ own learning, that differences in generative AI types (paid vs free versions) may create disparities in student outcomes leading to unfairness, and that confidential and personal information may be unintentionally leaked or disclosed. A survey of Japanese medical students [14] found that while 41.9% had experience using ChatGPT, only 10.2% had used it for medical assignments and 47% held negative views about its use for medical reports. Many students felt that, considering the time required to verify AI responses, independent learning would be more efficient, highlighting the essential need to cultivate critical evaluation skills for LLM outputs. A narrative review on chatbot integration in nursing education [15] also emphasizes the importance of ethical considerations, indicating the urgent need to establish ethical frameworks for AI use across nursing education.

The results of this study revealed that while LLMs demonstrated high accuracy rates on the Public Health Nurse National Examination, performance declined on multiple-choice questions. This finding has important implications for using LLMs as learning support tools in public health nursing education. Given the demonstrated limitations of LLMs in complex judgment requiring simultaneous consideration of multiple factors, LLMs are suitable for supplementary roles such as confirming foundational knowledge and gathering information, whereas faculty instruction remains crucial for learning scenarios requiring complex judgment. The Ministry of Education, Culture, Sports, Science and Technology has issued guidelines [13] on the educational use of generative AI at universities and colleges of technology, and public health nurse training institutions are also called upon to develop guidelines that clearly specify appropriate use scenarios and limitations for LLMs. A nationwide survey on information and communications technology (ICT) use among public health nurses in local governments [16] found that 82.8% responded that they did not know the procedures for promoting ICT use, indicating challenges in adapting to digital technology even among practicing public health nurses. This suggests the importance of providing systematic digital literacy education from the public health nurse training stage.

A detailed examination is needed regarding curriculum integration and faculty training. For example, development of specific implementation strategies is required, including in which courses and how LLMs should be introduced, how to design a phased introduction process, and how faculty should learn appropriate LLM use methods. In particular, establishing organizational training systems for enhancing faculty AI literacy and developing assessment methods premised on LLM use are important future challenges. To accumulate knowledge regarding these strategies, pilot program implementation and evaluation will be necessary.

As practical implications, there is potential for the use of LLMs as continuing education and self-directed learning support tools for practicing public health nurses. A concept analysis of LLMs in nursing education [17] positions LLMs as transformative tools that provide accessible and personalized learning support and promote cognitive and skill development. In public health nursing education as well, use is anticipated for responding to new public health issues and during information gathering stages in community diagnosis. Additionally, there is potential for the use of LLMs as an auxiliary tool in situations requiring rapid information organization, such as during disasters or emerging infectious disease outbreaks. However, a survey on ICT use among public health nurses in local governments [16] found that 89.1% of municipalities expressed concerns about individuals who have difficulty adapting to digital technology, necessitating careful introduction that considers the essence of interpersonal support in public health nursing work. Furthermore, while 55.9% in the same survey actively promoted ICT use, only 26.7% perceived progress as smooth, indicating challenges in digital literacy education for practicing public health nurses and establishing organizational support systems. As noted in the aforementioned concept analysis [17], careful attention must be paid to LLM limitations and ethical implications, ensuring that LLM integration aligns with the values and goals of nursing education. Therefore, when using LLMs in practical settings, it is essential to critically evaluate LLM outputs and integrate them with community characteristics and practical knowledge, considering the limitations in complex judgment revealed in this study.

The findings of this study occupy an important position within the broader context of AI use in health profession education. A narrative review on chatbots in nursing education [15] demonstrated a surge in related research from 2021 to 2023 (with 2023 accounting for 70% of publications), indicating growing scholarly interest in this field. Together with LLM evaluation studies on medical licensing examinations [4,5], it is becoming increasingly clear that LLMs demonstrate high performance across medical licensing examinations generally. However, the performance decline on multiple-choice questions demonstrated in this study indicates the existence of LLM limitations in complex judgment unique to public health nursing, such as integration of social determinants of health and planning of community-level interventions. This is consistent with findings from previous studies in medical education [10,11] showing that collective student judgment far exceeds that of AI, supporting the appropriate positioning of LLMs as educational and learning support tools rather than replacements for human health professionals. Additionally, a survey of medical students [14] showed that 47% held negative views about LLM use for medical assignments, indicating the recognition of the need for verification. A concept analysis in nursing education [17] also pointed out the need for careful consideration of LLM limitations and ethical implications. Furthermore, the aforementioned review on chatbot integration in nursing education [15] emphasizes the importance of ethical considerations and the urgency of original research while acknowledging it as a promising field. Collectively, these findings suggest that across health profession education generally, while LLMs have potential as useful auxiliary tools, cultivating the ability to understand their limitations and use them critically is a common challenge across all types of health profession education.

### Limitations and Future Directions

This study has the following strengths. First, this is the first study to evaluate the performance of multiple LLMs on the Public Health Nurse National Examination. While LLM evaluations have been conducted on medical and nursing licensing examinations, this study represents the first systematic evaluation in the public health nursing field. Second, reproducibility was ensured through the use of standardized prompts. Six prompts were created according to question types, achieving consistent evaluation. Third, detailed analysis by question format revealed the important finding of performance decline on multiple-choice questions. This discovery demonstrates LLM limitations in complex reasoning requiring the simultaneous consideration of multiple factors, with important implications for future educational implementation.

This study has several limitations. First, as a cross-sectional evaluation of a single year, temporal changes in LLM performance and reproducibility across different examination years could not be evaluated in this study. Second, accuracy rates alone do not clarify the quality of reasoning processes or correlation with actual public health nursing practice competencies. Third, while LLM versions and settings may influence results, this study was limited to evaluation using specific versions (GPT-4o, Claude Opus 4, and Gemini 2.5 Pro). Fourth, results may vary depending on prompt expression methods, and there is no guarantee that the standardized prompts used in this study are optimal. Fifth, because the overall mean score for examinees is not publicly available, direct performance comparison between LLMs and human public health nurse examinees is difficult. Furthermore, this study verified LLM performance in the educational evaluation context of a national examination and did not evaluate their utility or safety as decision support tools in actual public health nursing practice. Future research should include continued evaluation over multiple years, qualitative analysis of reasoning processes, validation of utility in practical settings, comparative studies with human public health nurses, and examination of applicability to decision support in actual practice.

### Conclusions

The LLMs evaluated demonstrated high performance on the Public Health Nurse National Examination; however, they also had limitations in solving problems requiring complex judgment. These findings provide important foundational data showing the possibilities and challenges of AI use in public health nursing. On the basis of these results, LLMs should be cautiously used as supplementary tools in public health nursing education.

## Abbreviations

AI: artificial intelligence
ICT: information and communications technology
LLM: large language model
